# Blood biomarkers in the application of diagnosis and prediction of overall survival for 1089 patients with nasopharyngeal carcinoma

**DOI:** 10.1038/s41598-023-42216-9

**Published:** 2023-09-12

**Authors:** Hangjiu Su, Yu Luo, Yanyun Chen, Zhongyuan Lin, Xiafei Fu, Songshan Zhu, Jun Yin

**Affiliations:** 1 Department of Laboratory Medicine, The People′s Hospital of Guangxi Zhuang Autonomous Region, Nanning, P.R. China; 2 Department of Pediatrics, The People′s Hospital of Guangxi Zhuang Autonomous Region, Nanning, P.R. China; 3 Guangdong Medical University, Dongguan, P.R. China; 4 Transplant Medical Center of The Second Affiliated Hospital of Guangxi Medical University, Guangxi Clinical Research Center for Organ Transplantation, Guangxi Key Laboratory of Organ Donation and Transplantation, Nanning, P.R. China

**Keywords:** Biochemistry, Cancer, Biomarkers, Diseases

## Abstract

Previous studies have indicated that some blood metrics play a crucial role in the diagnostic and prognostic values of various solid tumours. However, their comprehensive and unbiased comparison for nasopharyngeal carcinoma (NPC) has not been performed. Twenty blood metrics evaluated in tumours or noncancerous diseases were selected. We selected 1089 patients with NPC and analyzed the relationship between these metrics, clinical characteristics, and overall survival (OS). The albumin and prognostic nutritional index (PNI) exhibited a high area under the curve (AUC) value (> 0.7) together with high “sensitivity (Sen) + specificity (Spe) (> 1.5)” or Youden index (> 0.5) when compared to healthy populations. In comparing NPC and nasal polyps, 9 of 20 blood metrics showed a high AUC value (> 0.7). However, only the PNI and international normalised ratio show a sufficiently high Sen + Spe or Youden Index. None of them could distinguish the status of the TNM classification well. Only the lymphocyte-to-monocyte ratio (LMR) could predict the OS of patients with NPC (cut-off, 4.91; *p* = 0.0069). Blood metrics as non-invasive biomarkers are valuable tools for clinical management. Among these indicators, PNI is the most ideal indicator to distinguish NPC from healthy and nasal polyps. The LMR has good prognostic value.

Nasopharyngeal carcinoma (NPC) is a malignant tumour that originates in the nasopharyngeal epithelium. New cases and deaths for global NPC in 2020 were 133,354 and 80,008, respectively^1^. Compared to other cancer types, NPC is uncommon and shows a unique pattern of geographical distribution. There is a high incidence in some places, including South China, Southeast Asia, the Arctic region, North Africa, and parts of the Middle East^2^. Moreover, the occurrence of NPC is closely related to hereditary and viral risk factors, such as the Epstein-Barr virus. Early diagnosis of NPC is difficult and 60–70% of patients with the disease are diagnosed at an advanced stage (stages III and IV). The 5-year survival rates of stages I and II can exceed 60%. Thus, reliable biomarkers from the blood or other body fluids may be a promising alternative.

It has become evident that cancer-related inflammatory responses and the coagulation process create a good tumour microenvironment for tumourigenesis, invasion, and metastasis of cancer cells^3,4^. A number of studies have argued that inflammation-related indexes based on routine blood tests can be predictive factors in the diagnosis and prognosis of many different tumours or non-neoplastic diseases, such as the neutrophil-to-lymphocyte ratio (NLR). However, some researchers have questioned the reproducibility and reliability of inflammatory cell-based scores in clinical practice. For example, because of the variability of reported prognostic thresholds of NLR, platelet-to-lymphocyte ratio (PLR), and lymphocyte-to-monocyte ratio (LMR), their application in routine clinical practice should be reconsidered^5^. To evaluate the potential role of blood-related indices in the diagnosis of NPC, we selected the most common composite ratios from the literature, which will be compared to each other (Table 1)^6–12^. Some of these, such as FFR and DFR, have not been evaluated in NPC.

**Table 1:** Systemic blood metrics-based composite ratios.

Metric	Calculation
PNI	Prognostic nutritional index. 10 × serum albumin level (g/dL) + 0.005 × total lymphocyte count (per mm^3^)
AAPR	Albumin (ALB): alkaline phosphatase (ALP)
WLR	White blood cell (WBC) count: lymphocyte count
LMR	Lymphocyte count: monocyte count
NLR	Neutrophil count: lymphocyte count
PLR	Platelet count: lymphocyte count
PPR	Platelet distribution width: platelet count
MPR	Mean platelet volume (MPV): platelet count
SII	Systemic immune-inflammation index. Platelet count × neutrophil count/lymphocyte count
dNLR	Derived (d) NLR: neutrophil count/ (WBC − lymphocyte count)
PIR	Prothrombin time (PT): international normalised ratio (INR)
FFR	Fibrinogen degradation product (FDP): fibrinogen (FIB)
DFR	D-dimer: FIB

There are some ways in which hematological parameters are significant in the diagnosis and prognosis of cancer, including inflammation and immune response monitoring, tumor burden and stage determination, treatment monitoring and side effects, prognostic indicators, recurrence monitoring, and overall health assessment. It's important to note that hematological parameters can offer valuable information, they are one piece of the puzzle in diagnosing and managing NPC. Therefore, it is a promising aspect to comprehensively understand these parameters. The purpose of this study is to determine which blood-related indices are most suitable for the diagnosis and prediction of NPC.

## Material and methods

### Patients’ selection

A total of 1,089 patients (784 males and 305 females) with NPC were identified from a maintained database in a single surgical unit at the People’s Hospital of the Guangxi Zhuang Autonomous Region. Please note that all patients included in this study were diagnosed between January 2013 and January 2020 and had not received any treatment before diagnosis. The diagnosis was confirmed by histopathology. Patients who met the following criteria were included: 1. patients who underwent measurement of coagulation function and blood cell count within 1 week before surgery; 2. the exclusion criteria were haematologic diseases, other tumours, deep venous thrombosis, surgery, and diseases affecting coagulation and blood cell counts; 3. no drugs that affect blood metrics, such as warfarin, had been taken in the last 3 months. Patients were classified according to the TNM (tumour size, lymph node, metastasis) American Joint Committee of Cancer clinical staging algorithm, as revised by the 2008 Chinese Mainland Staging System Revision. A total of 130 healthy subjects (85 males and 45 females) and 119 patients (82 males and 37 females) with nasal polyps were randomly selected from the physical examinations as the control group. The People’s Hospital of the Guangxi Zhuang Autonomous Region has approved the experiments, including any relevant details. The experiments were conducted in compliance with the appropriate guidelines and regulations. Informed consent was obtained from all participants and/or their legal guardians.

### Blood metrics detection

Venous blood was collected into tubes with or without anticoagulants (109 mmol/L sodium citrate or EDTA). All blood metrics were measured within 2 h, including ALB, ALP, PT, INR, FIB, FDP, D-dimer, and blood cell count. The composite ratios were then calculated using the formulas in Table 1. Finally, we investigated the relationship between these blood metrics and the clinical characteristics of NPC.

### Fellow up

Patients with NPC were followed up primarily via telephone and periodic hospital visits. A total of 119 patients with NPC were followed up. Overall survival (OS) was defined as the period from the initial diagnosis to the last follow-up or death. The median follow-up time among the patients was 82 months (range, 3–118 months).

### Statistical analysis

Statistical analyses were performed using GraphPad Prism v8.4.3. Unpaired comparisons were conducted using one-way ANOVA or the Kruskal–Wallis test with Tukey’s or Dunn’s multiple comparison test. Comparisons between two groups were conducted using unpaired Student’s *t*-test. Differences between ALB, PNI, AAPR, and other indicators and clinicopathological characteristics of the patients were assessed using Pearson's χ test. The cut-off for individual metrics was examined using receiver operating characteristic (ROC) curve analyses. The threshold values of these characteristics were based on the most prominent point on the ROC curve for ‘sensitivity (Sen)’ and ‘1-specificity (Spe)’. The optimal threshold values were defined using the Youden index (maximum (Sen + Spe − 1)) and were used in subsequent analyses.

The accuracy of each metric for distinguishing the NPC and healthy populations was compared using the area under the curve (AUC). In general, the AUC results were classified under five criteria: 1. AUC values between 0.5 and 0.6 were considered failures. 2. AUC values between 0.6 and 0.7 were considered poor. 3. AUC values between 0.7 and 0.8 were considered fair. 4. AUC values between 0.8 and 0.9 were considered good. 5. AUC values between 0.9 and 1 were excellent^13^. If the AUC value was < 0.50, or the *p* value was > 0.05, the diagnostic test had no discriminatory ability. In this context, we set the Sen, Spe, and Youden index to 0. 

### Ethics approval and consent to participate

Approval for all procedures was granted by the Ethics Committee of the People's Hospital of the Guangxi Zhuang Autonomous Region.

## Results

### Clinicopathological characteristics

**Figure 1:**
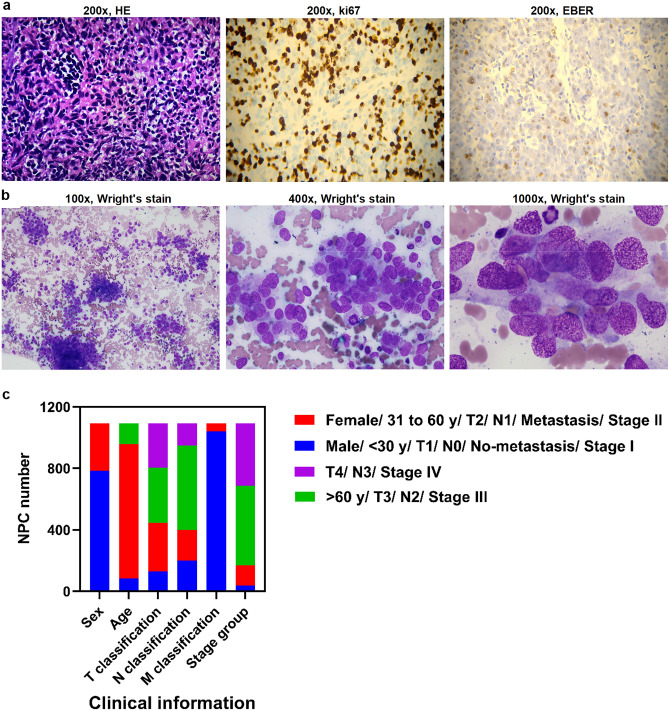
Clinicopathological characteristics. (**a**) Image presentation of tumor features, including morphology, expression of ki67, and EBER based on primary tumor location. (**b**) Image presentation of the metastasis of NPC cells into the bone marrow. (**c**) Main clinical characteristics of NPC patients.

Figure 1a,b show the morphology, high ki67 expression, and EBER positivity of NPC at the primary lesion. Moreover, it presents the morphology of NPC cells invading the bone marrow. A total of 1,089 patients were selected from the database. The majority of patients were men (72%), 30–60 years of age (80%). The tumour size of the majority of patients was > 5 cm (T3 and T4, approximately 59%). Most patients showed lymphovascular invasion (82%), but no distant organ metastasis (95%). Based on the clinical TNM stage and 5-year survival rate > 60%, we preliminarily defined stage I and II as an early group, whereas stages III and IV were defined as a late group. The majority of patients were in the late stage (III or IV, approximately 84%) (Fig. 1c).

### The association between clinical characteristics and blood metrics in patients with NPC

**Figure 2:**
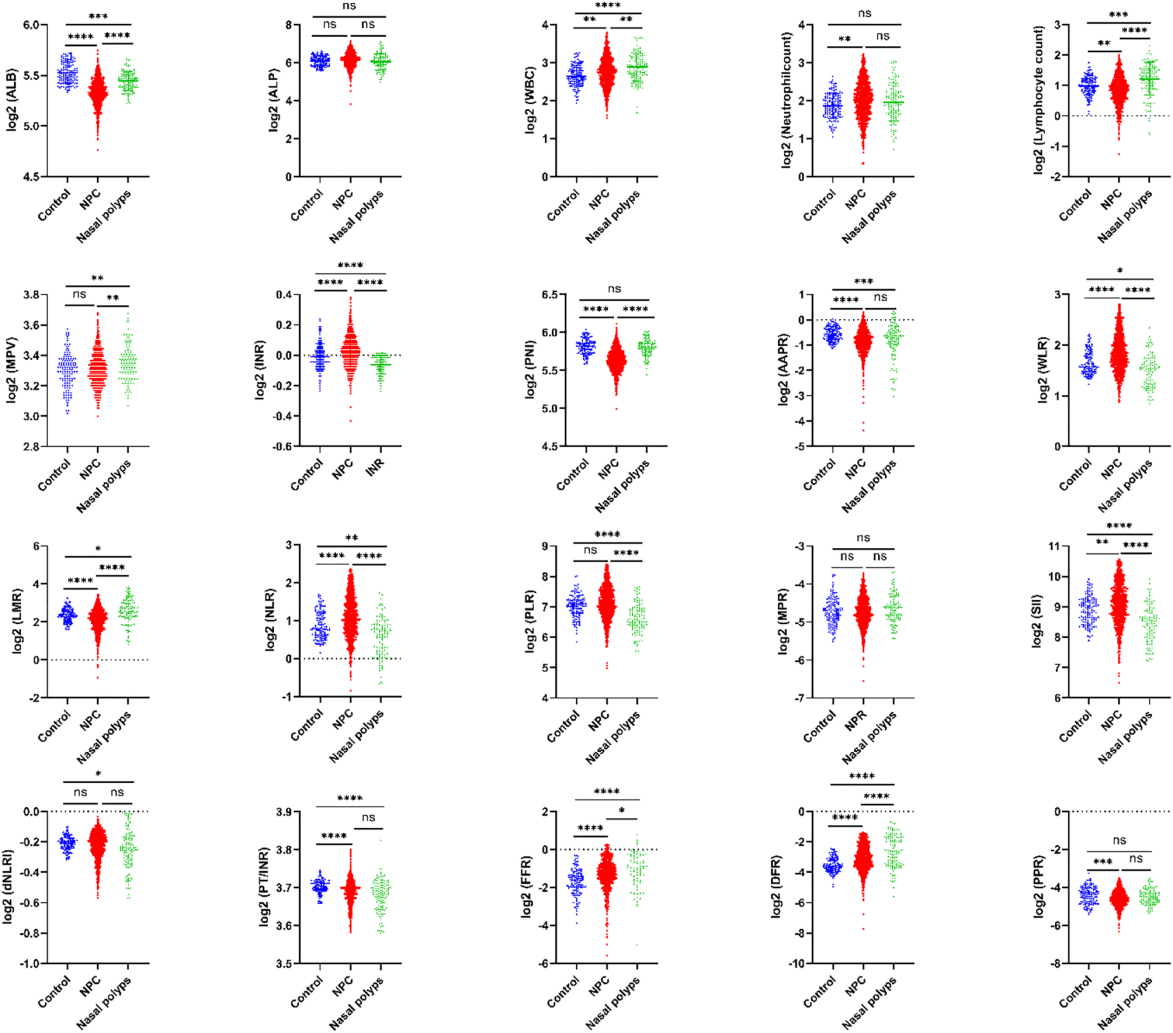
Quantitative presentation of the heterogeneity of blood metrics in the healthy population, NPC patients, and nasal polyps patients. Colors in scatter plots present patient type. Each symbol is a patient. Statistical significance between the groups was determined with a one-way ANOVA and Tukey’s multiple comparison test. The significance level for all tests is set at 0.05. The *p < 0.05, **p < 0.01, ***p < 0.001, ****p < 0.0001.

Nasal polyps, a benign proliferative disease, showed similar symptoms to NPC. It is important to compare the differences in their blood-related indices. Multiple comparison analyses among the three cohorts showed significant differences in blood metrics (Fig. 2). For example, patients with NPC are characterized by reduced ALB concentrations and elevated WLR. To further investigate the predictive value of these metrics for NPC, we performed ROC analysis, including patients with NPC versus healthy populations and patients with NPC versus patients with nasal polyps.

### Characteristics of blood metrics of NPC compared with normal controls

**Figure 3:**
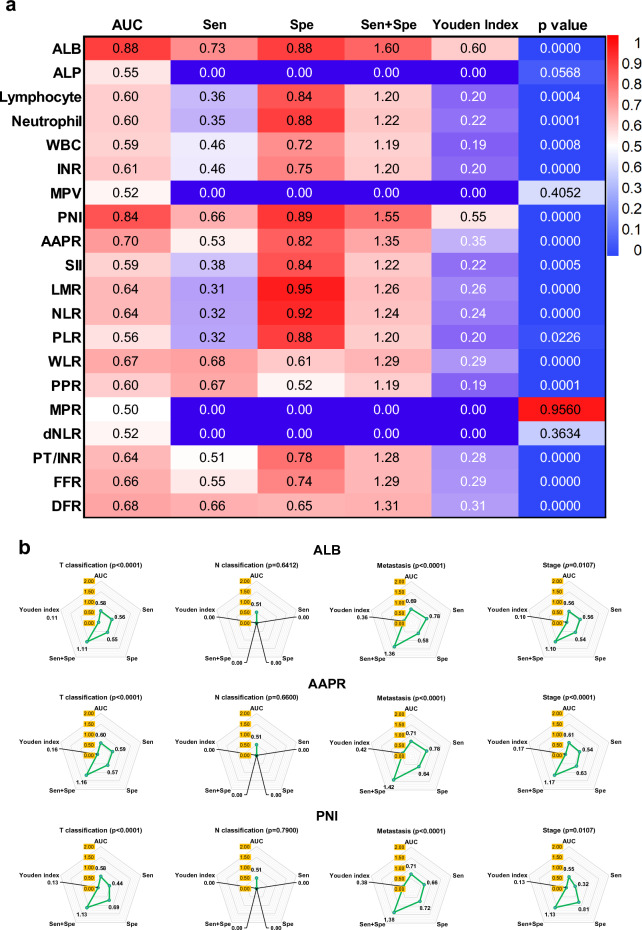
Characteristics of blood metrics of NPC compared with normal controls. (**a**) Comparison of the area under the ROC curve of blood metrics between NPC and healthy control. The number on the heat map indicated the value of AUC, sensitivity, specificity, Sen + Spe, Youden Index, and *p* value. If the AUC value is < 0.50 or the *p* > 0.05, the Sen, Spe, and Youden index are 0. (**b**) Comparison of the capacity of ALB, PNI, and AAPR in distinguishing the NPC occurrence and TNM classification. These values come from the ROC analysis and are shown in the spider plot. AUC, Sen, Spe, and Youden Index are closer to 1 means higher capacity. Sen + Spe is closer to 2 means higher capacity. When the *p* > 0.05, the metric is considered no distinguishing capacity and all indicators are recognized as 0.

ALB and PNI showed good predictive power for distinguishing patients with NPC from healthy populations. The APPR showed fair predicted power. Lymphocytes, neutrophils, INR, LMR, NLR, WLR, PPR, PT/INR, FFR, and DFR showed poor predictive power, whereas others showed no predictive power (Fig. 3a, Supplementary Fig. S1 online). Moreover, two rules of thumb related to the Sen, Spe, and Youden indices can be used as evidence to further explain test performance^14^.

1. For the test to be useful, Sen + Spe should be at least 1.5 (range of 0–1 is useless and 2 is perfect), or
2. If the Youden index is < 0.5, the test does not meet the empirical benchmarks for diagnostic purposes.

Therefore, only ALB and PNI can achieve an ideal effect on the test performance. We selected the metrics of AUC ≥ 0.7 for further analysis. The AUC of ALB, PNI, and APPR was significantly larger than that of the other metrics (AUC ≥ 0.7), indicating that they had better predictive values for patients with NPC.

The chi-square test was used to analyse the correlation between ALB, PNI, and APPR and clinicopathological parameters, inflammation metrics, and coagulation metrics. Patients with NPC were divided into different groups based on their cut-off values, which were derived from the comparison of NPC and healthy populations. ALB, PNI, and APPR significantly influenced these metrics. For example, low ALB, PNI, and APPR might have less distant organ invasion, but larger tumour size, more lymphovascular invasion, and advanced stage of the disease. Furthermore, these three metrics showed a similar relationship with the inflammation indices. For example, there was a significant positive correlation between NLR and a negative correlation with WLR. However, only ALB showed a higher correlation with coagulation indices (Table 2).

**Table 2:** The correlation between ALB, PNI, APPR, and NPC characteristics.

Clinical parameter	ALB ≤ 42.6	ALB > 42.6	χ^2^	*p*	PNI ≤ 51.9	PNI > 51.9	χ^2^	*p*	AAPR ≤ 0.57	AAPR > 0.57	χ^2^	*p*
Sex			5.60	**0.0180**			10.29	**0.0013**			20.29	** < 0.0001**
Male	557	226			491	292			448	335		
Female	240	66			224	82			128	178		
Age (year)			17.74	** < 0.0001**			17.03	** < 0.0001**			21.75	** < 0.0001**
≤ 60	679	277			606	350			480	476		
> 60	118	15			109	24			96	37		
Tumour size			9.53	**0.0020**			11.11	**0.0009**			23.04	** < 0.0001**
≤ 5 cm	303	142			266	179			196	249		
> 5 cm	494	150			449	195			380	264		
Lymphovascular invasion			1.73	0.1888			0.1711	0.6791			0.83	0.3636
No	137	61			133	65			111	87		
Yes	660	231			582	309			465	426		
Metastasis			7.01	**0.0081**			7.415	**0.0065**			15.10	**0.0001**
No	751	287			672	366			535	503		
Yes	46	5			43	8			41	10		
Stage group			12.11	**0.0070**			9.342	**0.0251**			23.65	** < 0.0001**
I	25	12			25	12			14	23		
II	88	44			85	47			55	77		
III	364	151			317	198			258	257		
IV	320	85			288	117			249	156		
Ki67 (n = 803)			0.03	0.8517			2.163	0.1414			1.32	0.2499
< 15%	37	15			28	24			24	28		
> 15%	551	200			488	263			416	335		
LMR (n = 1087)			10.41	**0.0013**			79.14	** < 0.0001**			254.20	** < 0.0001**
LMR ≤ 3.56	264	67			282	49			432	135		
LMR > 3.56	530	226			432	324			144	376		
NLR			7.70	**0.0055**			79.54	** < 0.0001**			8.16	**0.0043**
NLR ≤ 2.63	494	210			397	310			351	356		
NLR > 2.63	301	84			318	64			225	157		
WLR			11.21	**0.0008**			86.47	** < 0.0001**			6.03	**0.0141**
WLR ≤ 3.15	220	113			151	182			157	176		
WLR > 3.15	575	181			564	192			419	337		
PPR (N = 1047)			0.00	0.9802			4.766	**0.0290**			3.94	**0.0471**
PPR ≤ 0.05	617	220			478	271			465	372		
PPR > 0.05	154	56			212	86			100	110		
PT/INR (n = 1086)			16.31	** < 0.0001**			15.38	** < 0.0001**			1.98	0.1598
PT/INR ≤ 12.92	428	117			389	156			277	268		
PT/INR > 12.92	365	176			324	217			299	242		
FFR (822)			4.87	**0.0274**			2.16	0.1416			0.06	0.8020
FFR ≤ 0.40	265	112			240	137			216	161		
FFR > 0.40	344	101			306	139			250	195		
DFR (1062)			9.20	**0.0024**			3.039	0.0813			0.31	0.5753
DFR ≤ 0.09	220	110			223	137			196	164		
DFR > 0.09	555	177			474	228			368	334		

Significant values are in bold.

We compared the capacity of ALB, PNI, and AAPR to distinguish the NPC TNM classification, including tumour size (> 5 cm vs. < 5 cm), lymph node invasion, distant organ metastasis, and stage (early *vs.* late stage) (Fig. 3b). Interestingly, they showed different powers in predicting the prognosis of NPC. They all showed a high AUC value for NPC metastasis, together with relatively high Sen and Spe. APPR was a better metric than ALB and PNI for predicting the early and late stages. Although the sensitivity of PNI was lower than that of ALB and APPR, it can acquire higher specificity in predicting NPC occurrence, stage, tumour size, and organ metastasis. However, none of these metrics could predict lymph node invasion (*p* > 0.05). In addition, they had poor Youden index (< 0.5) and Sen + Spe (< 1.5) values.

### Characteristics of blood metrics of NPC compared with nasal polyps

**Figure 4:**
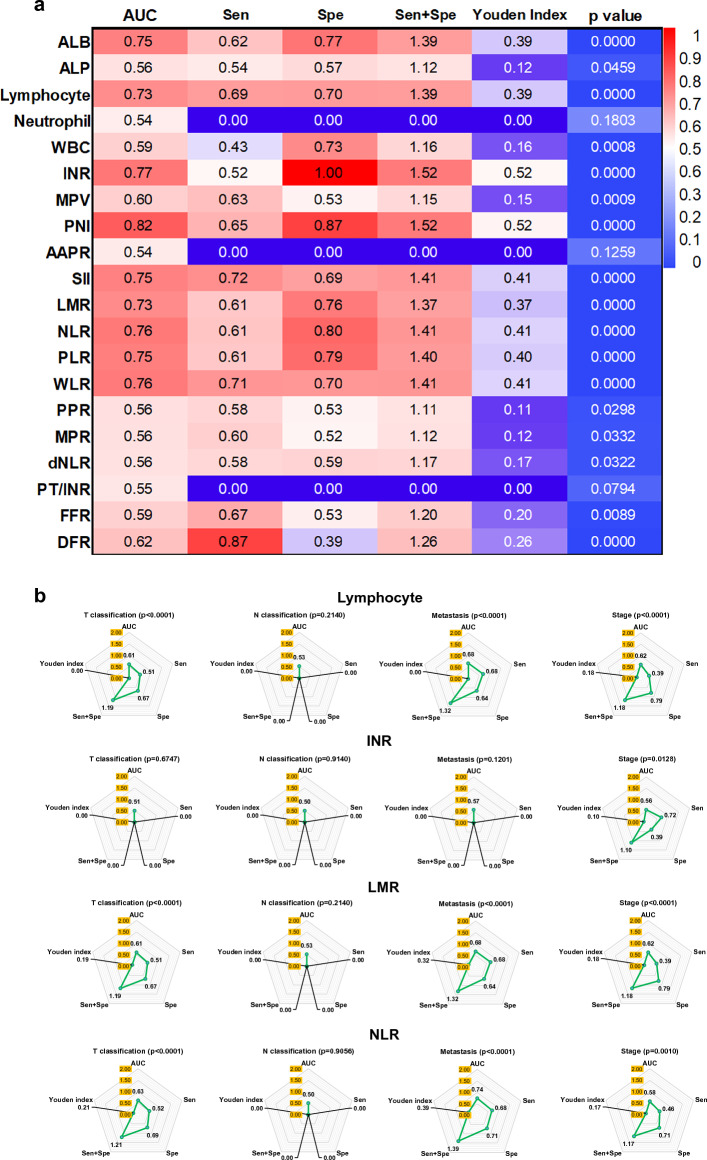

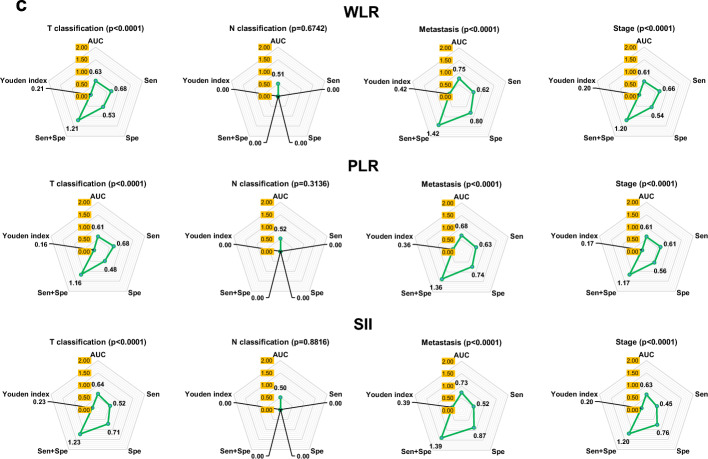
Characteristics of blood metrics of NPC compared with nasal polyps. (**a**) Comparison of the area under the ROC curve of blood metrics between NPC and nasal polyps. The number on the heat map indicated the value of AUC, sensitivity, specificity, Sen + Spe, Youden Index, and *p* value. If the AUC value is < 0.50 or the *p* > 0.05, the Sen, Spe, and Youden index are 0. (**b**) and (**c**) Comparison of the capacity of lymphocyte, INR, LMR, NLR, WLR, PLR, and SII in distinguishing the NPC occurrence and TNM classification. Panel A and panel B showed different metrics. These values come from the ROC analysis and are shown in the spider plot. AUC, Sen, Spe, and Youden Index are closer to 1 means higher capacity. Sen + Spe is closer to 2 means higher capacity. When the *p* > 0.05, the metric is considered no distinguishing capacity and all indicators are recognized as 0.

We used nasal polyps as controls to investigate the capacity of blood metrics to distinguish between NPC and nasal polyps. Many of them, such as ALB and PNI, showed high AUC values (> 0.7). However, few of them can show reasonable Sen + Spe (> 1.5) or Youden index (> 0.5) values, such as INR and PNI. Surprisingly, INR as a coagulation metric showed 100% Spe. Thus, these metrics with AUC values > 0.7 were selected to investigate their capacity to distinguish the TNM classification, including lymphocyte, INR, SII, LMR, NLR, PLR, and WLR (Fig. 4a, Supplementary Fig. S2 online). It is disappointing that none of the metrics can distinguish tumour size, lymph node invasion, organ metastasis, and disease stage. Even though the NRL, WLR, and SII had a high AUC value in predicting organ metastasis, the Sen + Sep or Youden index was not acceptable (Fig. 4b,c).

### Predictive capacity of blood metrics for OS

**Figure 5:**
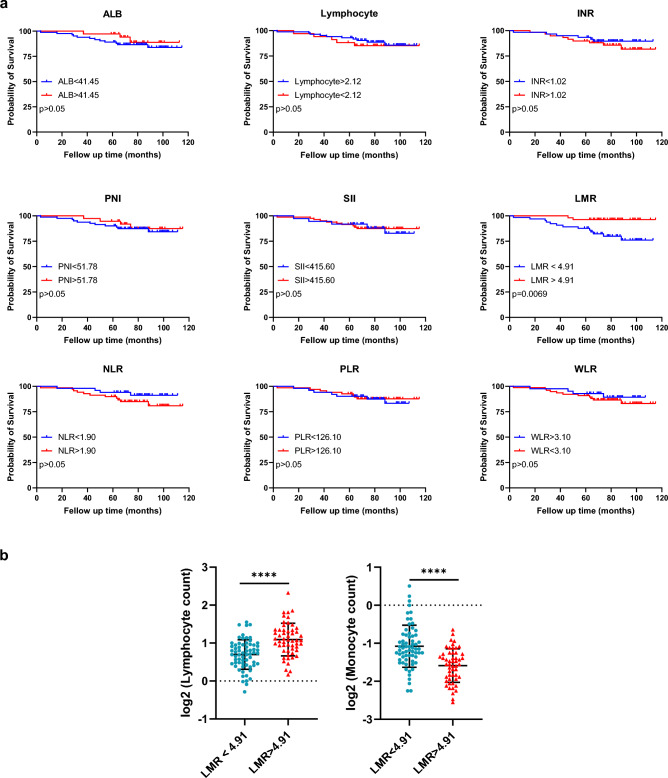
The comparison of predictive capacity of blood metrics for OS. (**a**) OS according to different blood metrics. The AUC of these metrics is larger than 0.7. Patients are grouped according to their cutoff. (**b**) Comparison of lymphocyte and monocyte count between NPC patients with LMR < 4.91 and NPC patients with LMR > 4.91. Colors in scatter plots present patient type. Each symbol is a patient. Statistical significance between the groups is determined with the unpaired Student's *t*-test. The significance level for all tests is set at 0.05. The ****p < 0.0001.

We selected blood metrics (AUC > 0.7) to investigate their prognostic value based on the comparison of NPC and nasal polyps, including ALB, lymphocyte number, INR, PNI, SII, LMR, NLR, PLR, and WLR. The patients were divided into two groups depending on the blood metric cut-off. Interestingly, most of these studies could not predict the OS of patients with NPC. Only patients grouped by LMR showed a significantly different OS. The cut-off LMR was 4.91. An LMR lower than the cut-off was associated with poor OS (*p* = 0.0069). Approximately 100 months after diagnosis, the OS of patients with a low LMR decreased to 75%, whereas patients with a high LMR maintained at least 90% OS (Fig. 5a). Compared to the group with an LMR < 4.91, the lymphocyte count was dramatically increased, but the monocyte count was significantly decreased in the group with an LMR > 4.91 (Fig. 5b).

## Discussion

There are different expression profiles of blood metrics between NPC, the healthy population, and nasal polyps. Our study found a high prevalence among young men. We demonstrated that most of them show a poor capacity to distinguish between NPC and normal populations. Only ALB, APPR, and PNI with high AUC values (> 0.7) could distinguish between the NPC and normal populations. ALB is the most abundant protein in the human blood circulation. The ALB concentration in NPC is significantly lower than that in healthy populations and nasal polyps. Tumours are characterised by larger fenestrations in the endothelium of the blood vessels and can disrupt the lymphatic system. This mechanism is responsible for the preferential accumulation of nanostructures and macromolecules, which is known as the enhanced permeability and retention effect (EPR)^15^. The ALB concentration in the blood (approximately 40 mg/mL) was significantly higher than that in the interstitium (approximately 14 mg/mL), which drives its diffusional transport. Thus, this transport power, together with the EPR effect, leads to the accumulation of ALB in the regions of proliferating tumours^16,17^. Another important mechanism is the strong endocytic capacity of tumour cells. They can utilise extracellular proteins as a source of amino acids to support tumour proliferation^18,19^. Therefore, these phenomena may explain why the ALB concentration in NPC decreases.

The AAPR was derived from ALB and ALP, which have prognostic value in patients with hepatocellular and lung cancers^7,20^. Some studies have indicated that ALP is highly expressed in patients with liver, colon, and breast cancers. A possible reason for this is the altered expression pattern of ALP isoenzymes in the liver, colon, and breast cancers. However, this was not the case for patients with NPC. Thus, the AUC value of AAPR was smaller than that of ALB but still reached 0.7. Different types of WBCs accumulate in the tumour microenvironment, such as neutrophils, lymphocytes, monocytes, and PLT. They can also contribute to tumour inhibition. However, tumour-associated inflammation is also favourable for all stages of tumour development^21^. In our previous research, we demonstrated that NPC can affect the expression of PLT and coagulation metrics, such as PT, FIB, FDP, and D-dimer^4^. In this study, most of the composite ratios of inflammation or coagulation showed different expression profiles in NPC, healthy populations, and nasal polyps. However, they cannot show enough distinction capacity between NPC and healthy populations.

PII is derived from ALB and lymphocytes, which are indicators of nutritional status and systemic inflammation in hepatocellular carcinoma and pancreatic cancer^22,23^. The lymphocyte counts and ALB levels of patients are significantly decreased. Thus, the PNI of NPC was also significantly decreased. However, the PNI can better distinguish NPC from healthy populations with high Sen + Spe and Youden index values. Of note, APPR and PII are derived from ALB. ALB is a better indicator than APPR and PII in distinguishing these situations, which is also reflected in the Sen + Spe and Youden indices. Low ALB, PNI, and APPR might lead to less organ invasion; however, patients have larger tumours and more advanced stages of the disease. However, they do not have a very close relationship with the TNM classification because of the low AUC, Sen + Spe, or Youden index. Taken together, ALB and PII are the best indicators for distinguishing patients with NPC from healthy individuals.

Nasal polyps are common noncancerous inflammatory diseases of the nasal and paranasal mucosa. It is a benign disease characterised by abundant extracellular fluid, mast cell degranulation, and infiltration of inflammatory cells, usually eosinophils. Occasionally, NPC is confused with nasal polyps. We demonstrated that most NPC blood metrics differ from nasal polyps. For example, the WLR is significantly increased in NPC, but the LMR is smaller. Interestingly, 9 out of 20 metrics with high AUC values could distinguish NPC from nasal polyps. In particular, the INR and PII show good Sen + Spe and Youden indices, respectively. INR is derived from PT and is an indicator for monitoring patients who are on warfarin, a vitamin K antagonist^24^. The INR is significantly increased in patients with NPC. In addition, INR shows very high Spe in distinguishing NPC from nasal polyps. Regrettably, none of them has a good capacity to identify tumour size, lymph node invasion, and distant organ metastasis.

The success rate of predicting patient survival for these single and composite ratios has reached disappointing heights, suggesting the limitations and lack of reliability in the application of prognostic value. We demonstrated that only the LMR has a prognostic value for patient survival. Tumour-infiltrating lymphocytes (TILs) and tumour-associated macrophages (TMAs) are important prognostic factors for various cancers^25^. TILs are thought to be responsible for controlling both cellular and humoral antitumour immune responses in tumours. High levels of TILs are associated with improved clinical outcomes^26^. Monocytes are the major source of TAMs and dendritic cells that shape the tumour microenvironment^27^. M2 macrophages related to wound healing and tissue repair can help tumour escape immune surveillance and is a “promoting tumour” phenotype^28^.

Some studies have shown that these metrics have prognostic value in solid cancers^8,10,29^, such as LMR, NLR, and AAPR. However, there were some differences in our study. The possible reasons include the following: 1. Heterogeneity of tumour types; 2. Heterogeneity of the cut-off; For example, Tomohiro et al*.* demonstrated the impact of different cut-off values on the prognostic effect of the LMR. We chose the cut-off value from the comparison between NPC and nasal polyps. However, the cut-off value in some studies is derived from the comparison between patients and healthy individuals; and 3. Standardisation varies. Some studies only focused on the prognostic value but ignored the distinguishing capacity (AUC > 0.7). In this study, we focused on several conditions. The first is the capacity to distinguish NPC from healthy populations or benign diseases, such as nasal polyps. The Youden index measures the ability of a diagnostic test to Sen (detect disease) and Spe (detect healthy or free of disease). Therefore, we further investigated their Sen + Spe and Youden indices. For these indicators with an AUC > 0.7, the prognostic values should be further studied. In our study, we sought to demonstrate that an indicator can discriminate diseases with high AUC values and predict clinical outcomes. Taken together, the LMR may be the best among the above studied blood metrics.

## Conclusion

In conclusion, our study broadly compared various blood metrics for the application of the diagnostic and prognostic values of NPC. The PNI is the most ideal metric for identifying NPC in healthy and nasal polyp populations. LMR has a good prognostic value. As non-invasive biomarkers, they are valuable tools for clinical management. In the future, we will compare the impact of the different cut-off values of these metrics on their diagnostic and prognostic values and validate their value.

### Supplementary Information

Supplementary Information.

## Data Availability

The datasets generated for this study are available on request to the corresponding author.
