# Supplementary material for: Blood biomarkers in the application of diagnosis and prediction of overall survival for 1089 patients with nasopharyngeal carcinoma
Source: Sci Rep. 2023 Sep 12;13:15082. doi: 10.1038/s41598-023-42216-9 (PMC10497678; doi:10.1038/s41598-023-42216-9)
Supplement: Supplementary file 1 — Supplementary Information. [file 41598_2023_42216_MOESM1_ESM.pdf]

Blood biomarkers in the application of diagnosis and prediction of overall survival for 1,089 patients with nasopharyngeal carcinoma

Hangjiu Su[2], Yu Luo[2], Yanyun Chen[2], Zhongyuan Lin[2], Xiafei Fu[3], Songshan Zhu[4*], Jun Yin[1*]

1. Transplant Medical Center of The Second Affiliated Hospital of Guangxi Medical University, Guangxi Key Laboratory of Organ Donation and Transplantation, Guangxi Transplantation Medicine Research Center of Engineering Technology, Guangxi, P.R. China.
2. Department of Laboratory Medicine, The People's Hospital of Guangxi Zhuang Autonomous Region, Guangxi, P.R. China.
3. Department of Pediatrics, The People's Hospital of Guangxi Zhuang Autonomous Region, Guangxi, P.R. China.
4. Guangdong Medical University, Dongguan, P.R. China..

Corresponding author:

Jun Yin, Email: miriam_yinjun@163.com;

Songshan Zhu, Email: zss21563@163.com

Supplementary Fig. S1

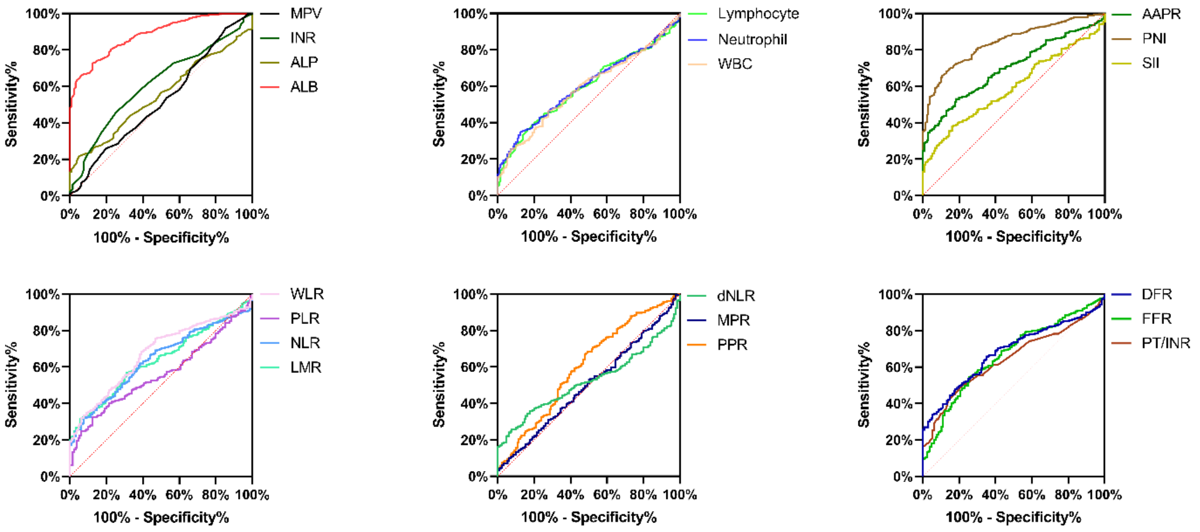

Supplementary Fig. S1 | ROC curve showed the diagnostic efficacy of blood metrics between NPC and healthy control. The red dotted line indicates the reference line. Other solid lines indicate the different blood metrics.

Supplementary Fig. S2

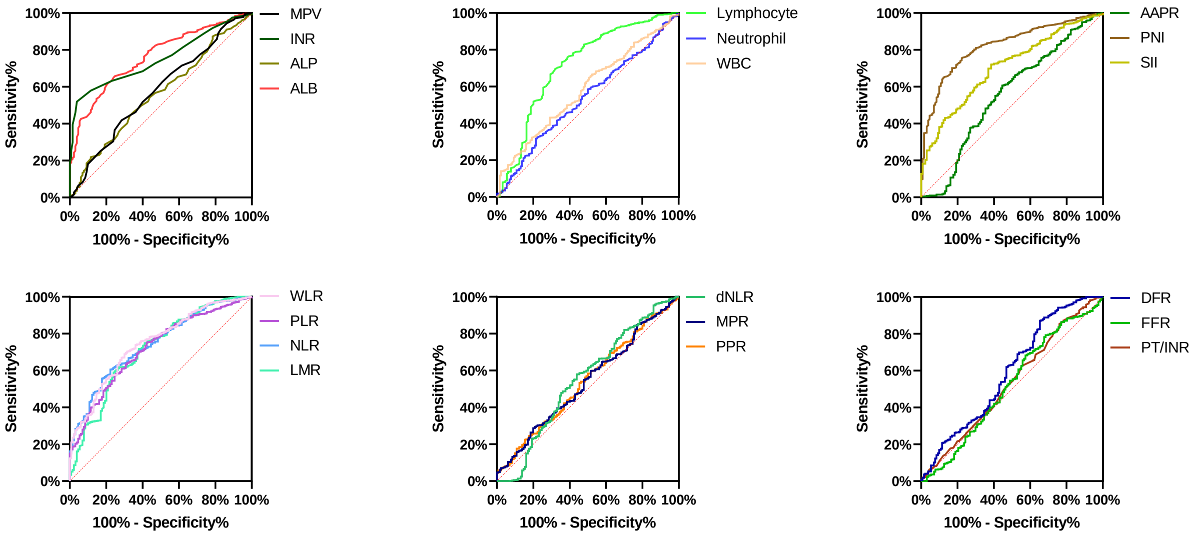

Supplementary Fig. S2 | ROC curve showed the diagnostic efficacy of blood metrics between NPC and nasal polyps. The red dotted line indicates the reference line. Other solid lines indicate the different blood metrics.
